# Supplementary material for: The NuRD complex cooperates with SALL4 to orchestrate reprogramming
Source: Nat Commun. 2023 May 18;14:2846. doi: 10.1038/s41467-023-38543-0 (PMC10199099; doi:10.1038/s41467-023-38543-0)
Supplement: Supplementary file 1 — Supplementary Information [file 41467_2023_38543_MOESM1_ESM.pdf]

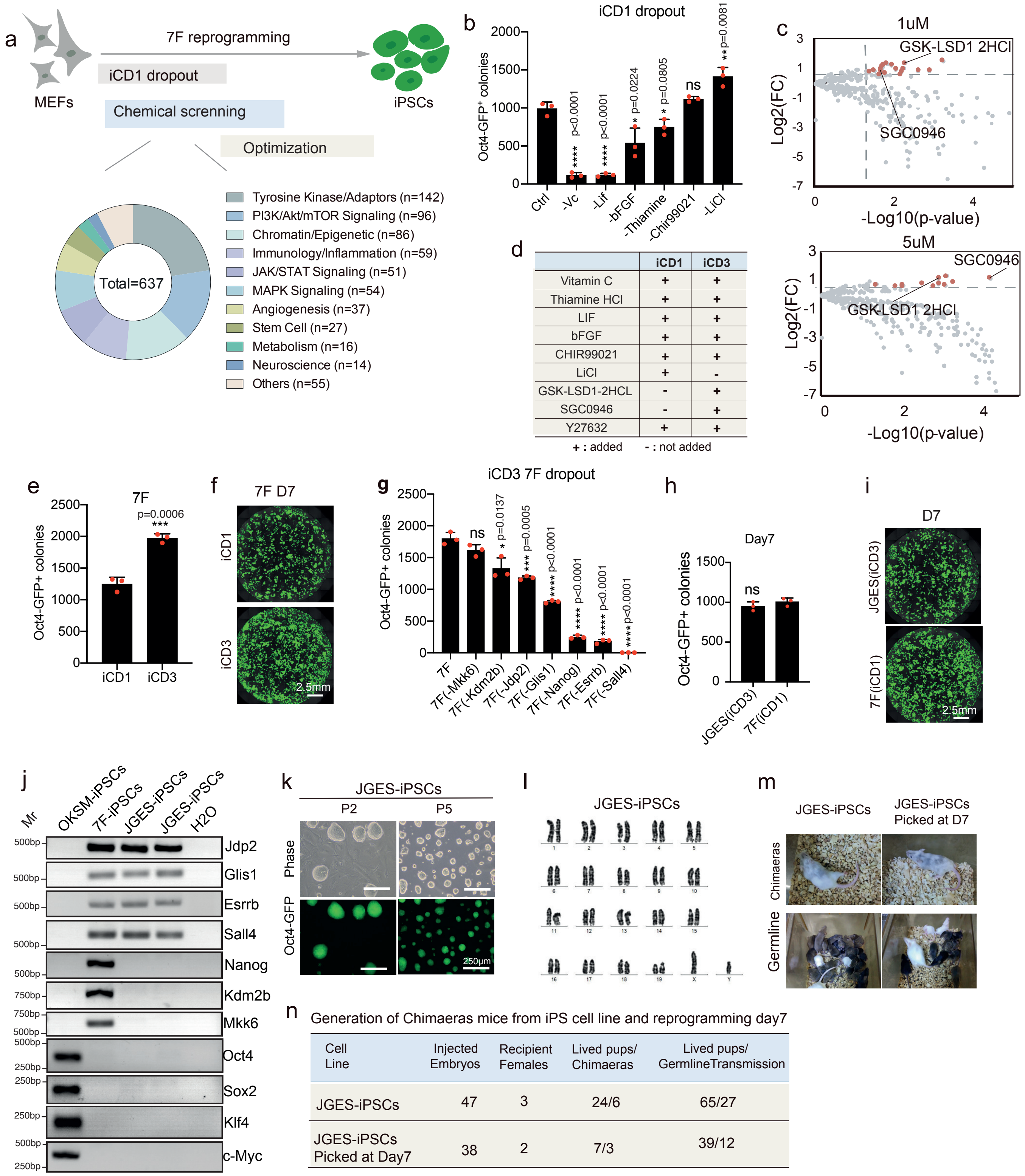
a
7F reprogramming
iCD1 dropout
Chemical screnning
Optimization
MEFs
iPSCs
Total=637
Tyrosine Kinase/Adaptors (n=142)
PI3K/Akt/mTOR Signaling (n=96)
Chromatin/Epigenetic (n=86)
Immunology/Inflammation (n=59)
JAK/STAT Signaling (n=51)
MAPK Signaling (n=54)
Angiogenesis (n=37)
Stem Cell (n=27)
Metabolism (n=16)
Neuroscience (n=14)
Others (n=55)
b
iCD1 dropout
Oct4-GFP+ colonies
Ctrl
-Vc
-Lif
-bFGF
-Thiamine
-Chir99021
-LiCl
**** p<0.0001
**** p<0.0001
* p=0.0224
* p=0.0805
ns
** p=0.0081
c
1uM
GSK-LSD1 2HCl
SGC0946
Log2(FC)
-Log10(p-value)
5uM
SGC0946
GSK-LSD1 2HCl
d
	iCD1	iCD3
Vitamin C	+	+
Thiamine HCl	+	+
LIF	+	+
bFGF	+	+
CHIR99021	+	+
LiCl	+	-
GSK-LSD1-2HCL	-	+
SGC0946	-	+
Y27632	+	+
+ : added   - : not added
e
7F
p=0.0006
***
Oct4-GFP+ colonies
iCD1
iCD3
f
7F D7
iCD1
iCD3
2.5mm
g
iCD3 7F dropout
Oct4-GFP+ colonies
7F
7F(-Mkk6)
7F(-Kdm2b)
7F(-Jdp2)
7F(-Glis1)
7F(-Nanog)
7F(-Esrrb)
7F(-Sall4)
ns
* p=0.0137
*** p=0.0005
**** p<0.0001
**** p<0.0001
**** p<0.0001
**** p<0.0001
h
Day7
ns
Oct4-GFP+ colonies
JGES(iCD3)
7F(iCD1)
i
D7
JGES(iCD3)
7F(iCD1)
2.5mm
j
Mr
OKSM-iPSCs
7F-iPSCs
JGES-iPSCs
JGES-iPSCs
H2O
Jdp2
Glis1
Esrrb
Sall4
Nanog
Kdm2b
Mkk6
Oct4
Sox2
Klf4
c-Myc
k
JGES-iPSCs
P2
P5
Phase
Oct4-GFP
250μm
l
JGES-iPSCs
m
JGES-iPSCs
JGES-iPSCs Picked at D7
Chimaeras
Germline
n
Generation of Chimaeras mice from iPS cell line and reprogramming day7
Cell Line	Injected Embryos	Recipient Females	Lived pups/ Chimaeras	Lived pups/ GermlineTransmission
JGES-iPSCs	47	3	24/6	65/27
JGES-iPSCs Picked at Day7	38	2	7/3	39/12

Supplementary Fig. 1 Establishment of JGES reprogramming

a, Schematic diagram of establishment of JGES(Jdp2-Glis1-Esrrb-Sall4)-iCD3 reprograming system. **b**, Single chemical dropout based on 7F-iCD1 system. Data are mean ± s.d., two-sided, unpaired t test; n = 3 independent experiments, $^*p < 0.05$, $^{**}p < 0.01$ $^{****}p < 0.0001$. **c**, GSK-LSD1 2HCl and SGC0946 hits at 1 μM and 5 μM concentration among the chemical screening library. Two-sided, unpaired t test; n = 3 independent experiments, FC(Fold Change) = 1.5, p-value < 0.05. **d**, Components and differences between iCD1 and iCD3. **e**, **f**, GFP+ clones number and photograph of 7F-iCD1 and 7F-iCD3 system at day7 per 1.5×10^4 MEF cells. Scale bars, 2.5mm. Data are mean ± s.d., two-sided, unpaired t test; n = 3 independent experiments, $^{***}p < 0.001$. **g**, Oct4-GFP+ colonies number of single factor dropout based on 7F-iCD3 at day7 per 1.5×10^4 MEF cells. Data are mean ± s.d., two-sided, unpaired t test; n = 3 independent experiments, $^*p < 0.05$, $^{***}p < 0.001$, $^{****}p < 0.0001$. **h**,**i**, Comparation between JGES and 7F GFP+ clones number at day7 in iCD3 medium. Scale bars, 2.5mm. Data are mean ± s.d., two-sided, unpaired t test; n = 3 independent experiments, ns > 0.05. **j**, PCR for genomic insertion identification of OKSM-iPSCs, 7F-iPSCs, JGES-iPSCs-1, JGES-iPSCs-2. **k**, Morphology of JGES-iPSCs at passage 2 and 5. Scale bars, 250 μm. **l**, Karyotype of JGES-iPSCs. **m**, Offspring of JGES-iPSCs cell line and JGES-iPSCs clones picked at day7 Chimeric and germline transmission mice. **n**, Table to show living pups of Chimaeras and germline transmission of JGES-iPSCs cell line and JGES-iPSCs clones picked at day7. Source data related to Fig 1b, e,g,h are provided as a Source Data file.

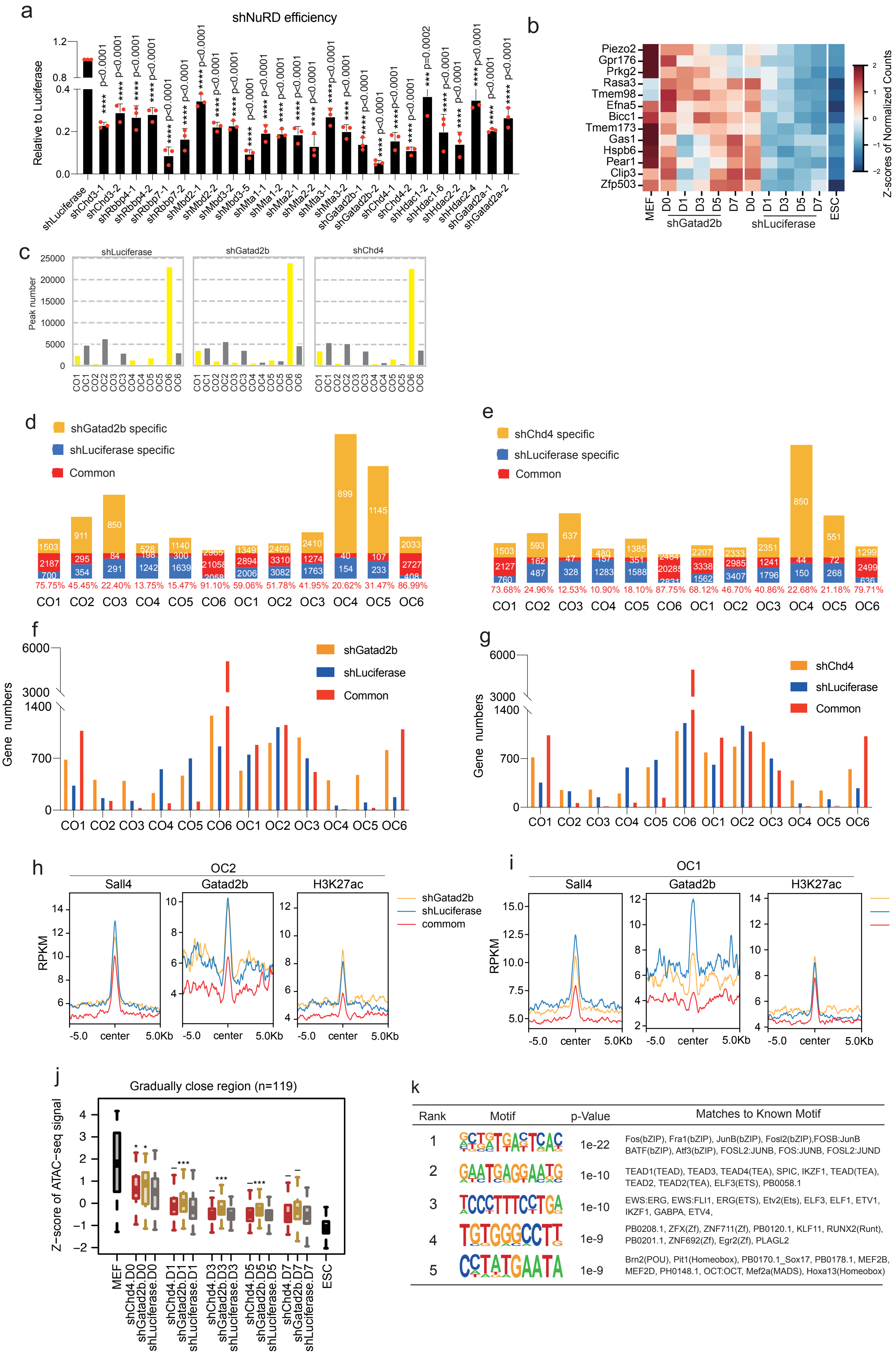
a
shNuRD efficiency
Relative to Luciferase
shLuciferase
shChd3-1
shChd3-2
shRbbp4-1
shRbbp4-2
shRbbp7-1
shRbbp7-2
shMbd2-1
shMbd2-2
shMbd3-2
shMbd3-5
shMta1-1
shMta1-2
shMta2-1
shMta2-2
shMta3-1
shMta3-2
shGatad2b-1
shGatad2b-2
shChd4-1
shChd4-2
shHdac1-2
shHdac1-6
shHdac2-2
shHdac2-4
shGatad2a-1
shGatad2a-2
b
Piezo2
Gpr176
Prkg2
Rasa3
Tmem98
Efna5
Bicc1
Tmem173
Gas1
Hspb6
Pear1
Clip3
Zfp503
MEF
D0
D1
D3
D5
D7
shGatad2b
shLuciferase
ESC
Z-scores of Normalized Counts
c
shLuciferase
shGatad2b
shChd4
Peak number
d
shGatad2b specific
shLuciferase specific
Common
CO1
CO2
CO3
CO4
CO5
CO6
OC1
OC2
OC3
OC4
OC5
OC6
e
shChd4 specific
shLuciferase specific
Common
f
shGatad2b
shLuciferase
Common
Gene numbers
g
shChd4
shLuciferase
Common
Gene numbers
h
OC2
Sall4
Gatad2b
H3K27ac
RPKM
shGatad2b
shLuciferase
commom
-5.0
center
5.0Kb
i
OC1
Sall4
Gatad2b
H3K27ac
RPKM
shChd4
shLuciferase
commom
j
Gradually close region (n=119)
Z−score of ATAC−seq signal
MEF
shChd4.D0
shGatad2b.D0
shLuciferase.D0
shChd4.D1
shGatad2b.D1
shLuciferase.D1
shChd4.D3
shGatad2b.D3
shLuciferase.D3
shChd4.D5
shGatad2b.D5
shLuciferase.D5
shChd4.D7
shGatad2b.D7
shLuciferase.D7
ESC
k
Rank
Motif
p-Value
Matches to Known Motif
1
1e-22
Fos(bZIP), Fra1(bZIP), JunB(bZIP), Fosl2(bZIP),FOSB:JunB
BATF(bZIP), Atf3(bZIP), FOSL2:JUNB, FOS:JUNB, FOSL2:JUND
2
1e-10
TEAD1(TEAD), TEAD3, TEAD4(TEA), SPIC, IKZF1, TEAD(TEA),
TEAD2, TEAD2(TEA), ELF3(ETS), PB0058.1
3
1e-10
EWS:ERG, EWS:FLI1, ERG(ETS), Etv2(Ets), ELF3, ELF1, ETV1,
IKZF1, GABPA, ETV4,
4
1e-9
PB0208.1, ZFX(Zf), ZNF711(Zf), PB0120.1, KLF11, RUNX2(Runt),
PB0201.1, ZNF692(Zf), Egr2(Zf), PLAGL2
5
1e-9
Brn2(POU), Pit1(Homeobox), PB0170.1_Sox17, PB0178.1, MEF2B,
MEF2D, PH0148.1, OCT:OCT, Mef2a(MADS), Hoxa13(Homeobox)

Supplementary Fig. 2 Knockdown NuRD subunits compromise JGES reprogramming

a, knocking down efficiency of NuRD subunits by shRNAs compared to shLciferase, n=3 independent replicates. Data are mean ± s.d., two-sided, unpaired t test; n = 3 independent experiments, ***p < 0.01 ****p < 0.0001. Source data is provided as a Source Data file. **b**, Heatmap showing the transcription of represented genes that failed to silence with knocking down Gatad2b. **c**, Bar plot showing the number of peaks defined in each OC/CO categories. Loci of open chromatin were arranged into groups depending upon the day of reprogramming they changed from closed to open (CO) or open to closed (OC) in shLuciferase. **d, e**, Bar plot showing the number of common and specific peaks in each OC/CO category when comparing shGatad2b (**d**) or shChd4 (**e**) to shLuciferase. Colors indicated peaks found in shGatad2b or shChd4 (yellow) only, shLuciferase (blue) only and both (red), respectively. Data under each column are presented as the percentage of common peaks that found in shGatad2b or shChd4 relative to shLuciferase. **f**, **g**, Bar plot showing the number of genes located from peaks in fig **d** and **e,** respectively. **h**, **i**, Pileup of Sall4, Gatad2b and H3K27ac CUT&Tag signal for specific and common regions in OC2 (shGatad2b) and OC1 (shChd4), respectively. **j**, Box plot showing the ATAC-seq signal of the gradually closing regions with knockdown Gatad2b and Chd4. The box plots indicate the medians (centerlines), first and third quartiles (bounds of boxes) and 1.5 multiply by interquartile range (whiskers). A two-sided Student's t-test was performed for comparisons between classes (***: p-value < 0.001; **: p-value < 0.01; *: p-value < 0.05; -: p-value > 0.05). **k**, Enriched motifs of the regions in **j**. Source data related to Fig s2a is provided as a Source Data file.

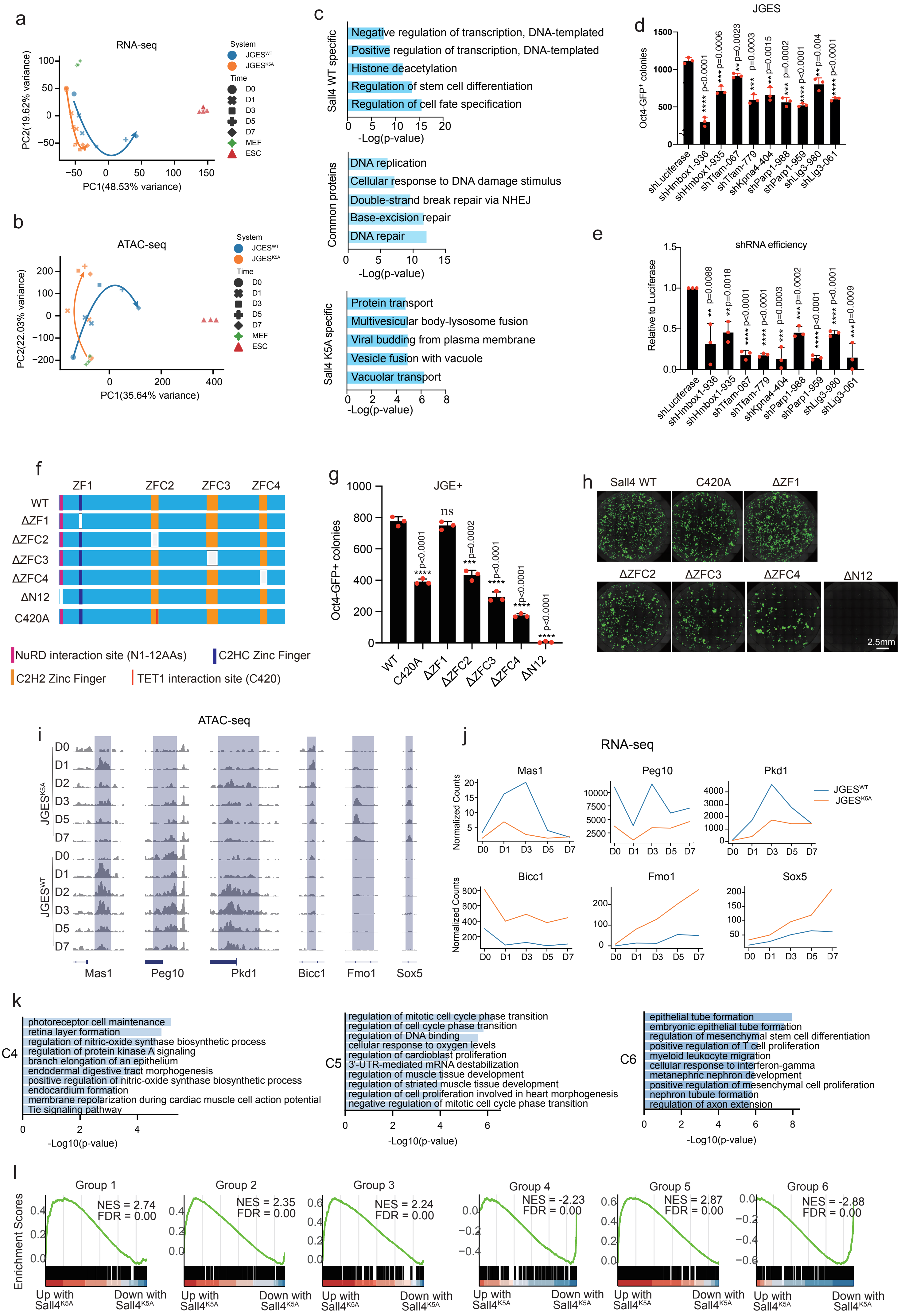
a
RNA-seq
PC2(19.62% variance)
PC1(48.53% variance)
System
JGES^WT
JGES^K5A
Time
D0
D1
D3
D5
D7
MEF
ESC
b
ATAC-seq
PC2(22.03% variance)
PC1(35.64% variance)
c
Sall4 WT specific
Negative regulation of transcription, DNA-templated
Positive regulation of transcription, DNA-templated
Histone deacetylation
Regulation of stem cell differentiation
Regulation of cell fate specification
-Log(p-value)
Common proteins
DNA replication
Cellular response to DNA damage stimulus
Double-strand break repair via NHEJ
Base-excision repair
DNA repair
Sall4 K5A specific
Protein transport
Multivesicular body-lysosome fusion
Viral budding from plasma membrane
Vesicle fusion with vacuole
Vacuolar transport
d
JGES
Oct4-GFP+ colonies
shLuciferase
shHmbox1-936
shHmbox1-935
shTfam-067
shTfam-779
shKpna4-404
shParp1-988
shParp1-959
shLig3-980
shLig3-061
e
shRNA efficiency
Relative to Luciferase
f
ZF1
ZFC2
ZFC3
ZFC4
WT
ΔZF1
ΔZFC2
ΔZFC3
ΔZFC4
ΔN12
C420A
NuRD interaction site (N1-12AAs)
C2HC Zinc Finger
C2H2 Zinc Finger
TET1 interaction site (C420)
g
JGE+
Oct4-GFP+ colonies
ns
h
Sall4 WT
C420A
ΔZF1
ΔZFC2
ΔZFC3
ΔZFC4
ΔN12
2.5mm
i
ATAC-seq
JGES^K5A
JGES^WT
Mas1
Peg10
Pkd1
Bicc1
Fmo1
Sox5
j
RNA-seq
Normalized Counts
k
C4
photoreceptor cell maintenance
retina layer formation
regulation of nitric-oxide synthase biosynthetic process
regulation of protein kinase A signaling
branch elongation of an epithelium
endodermal digestive tract morphogenesis
positive regulation of nitric-oxide synthase biosynthetic process
endocardium formation
membrane repolarization during cardiac muscle cell action potential
Tie signaling pathway
C5
regulation of mitotic cell cycle phase transition
regulation of cell cycle phase transition
regulation of DNA binding
cellular response to oxygen levels
regulation of cardioblast proliferation
3'-UTR-mediated mRNA destabilization
regulation of muscle tissue development
regulation of striated muscle tissue development
regulation of cell proliferation involved in heart morphogenesis
negative regulation of mitotic cell cycle phase transition
C6
epithelial tube formation
embryonic epithelial tube formation
regulation of mesenchymal stem cell differentiation
positive regulation of T cell proliferation
myeloid leukocyte migration
cellular response to interferon-gamma
metanephric nephron development
positive regulation of mesenchymal cell proliferation
nephron tubule formation
regulation of axon extension
-Log10(p-value)
l
Enrichment Scores
Group 1
NES = 2.74
FDR = 0.00
Group 2
NES = 2.35
FDR = 0.00
Group 3
NES = 2.24
FDR = 0.00
Group 4
NES = -2.23
FDR = 0.00
Group 5
NES = 2.87
FDR = 0.00
Group 6
NES = -2.88
FDR = 0.00
Up with Sall4^K5A
Down with Sall4^K5A

Supplementary Fig. 3 Sall4 mutation and deletion inhibits JGES reprogramming

a, PCA of transcriptome dynamics during JGES reprogramming by WT or K5A mut Sall4 in combination with JGE. Blue and yellow solid line with arrows represent WT and K5A mut Sall4 respectively. **b**, PCA of chromatin accessibility during reprogramming by WT or K5A mut Sall4 in combination with JGE. Blue and yellow solid line with arrows represent WT and K5A mut Sall4 respectively. **c**, Gene ontology analysis of IP-MS enriched proteins. **d**, Oct4-GFP positive colonies number of depletion the genes of $Sall4^{WT}$ and $Sall4^{K5A}$ common enriched proteins by shRNA during JGES reprogramming. Data are mean ± s.d., two-sided, unpaired t test; n = 3 independent experiments, **p < 0.01, ***p < 0.001, ****p < 0.0001. **e**, Knockdown efficiency of the genes of $Sall4^{WT}$ and $Sall4^{K5A}$ common enriched proteins by shRNA; Data are mean ± s.d., two-sided, unpaired t test; n = 3 independent experiments, **p < 0.01, ***p < 0.001, ****p < 0.0001. **f,** Diagram of mutation and truncated SALL4 protein. **g, h**, OCT4-GFP positive colonies number and photograph of mutation and truncated Sall4 co-transfected with Jdp2-Glis1-Esrrb at day7. Scale bars, 2.5mm. Data are mean ± s.d., two-sided, unpaired t test; n = 3 independent experiments, ns > 0.05. ***p < 0.001, ****p < 0.0001. **i**, Selected genomic views of the ATAC-seq data: Mas1 (chr17:12,838,069-12,851,859, scale: 0-50 RPKM), Peg10 (chr6:4,747,306-4,760,517, scale: 0-50 RPKM), Pkd1 (chr17:24,549,950-24,596,514, scale: 0-50 RPKM), Bicc1 (chr10:70,922,837-71,159,690, scale: 0-70 RPKM), Fmo1 (chr1:162,829,788-162,860,002, scale: 0-150 RPKM), Sox5 (chr6:143,828,425-143,947,108, scale: 0-100 RPKM). Identified regions are marked with a gray box. **j**, Line plot showing the expression for the respective genes is shown in i. **k**, gene ontology (GO) analysis of the genes located in the region cluster in 2f. **l**, Gene-set enrichment analysis of the gene clusters in Fig 1f to the samples in reprogramming of WT/K5A Sall4 with JGE on day3. NES, normalized enrichment score; FDR, false discovery rate. Source data related to Fig s3d,e,g are provided as a Source Data file.

Fig S4

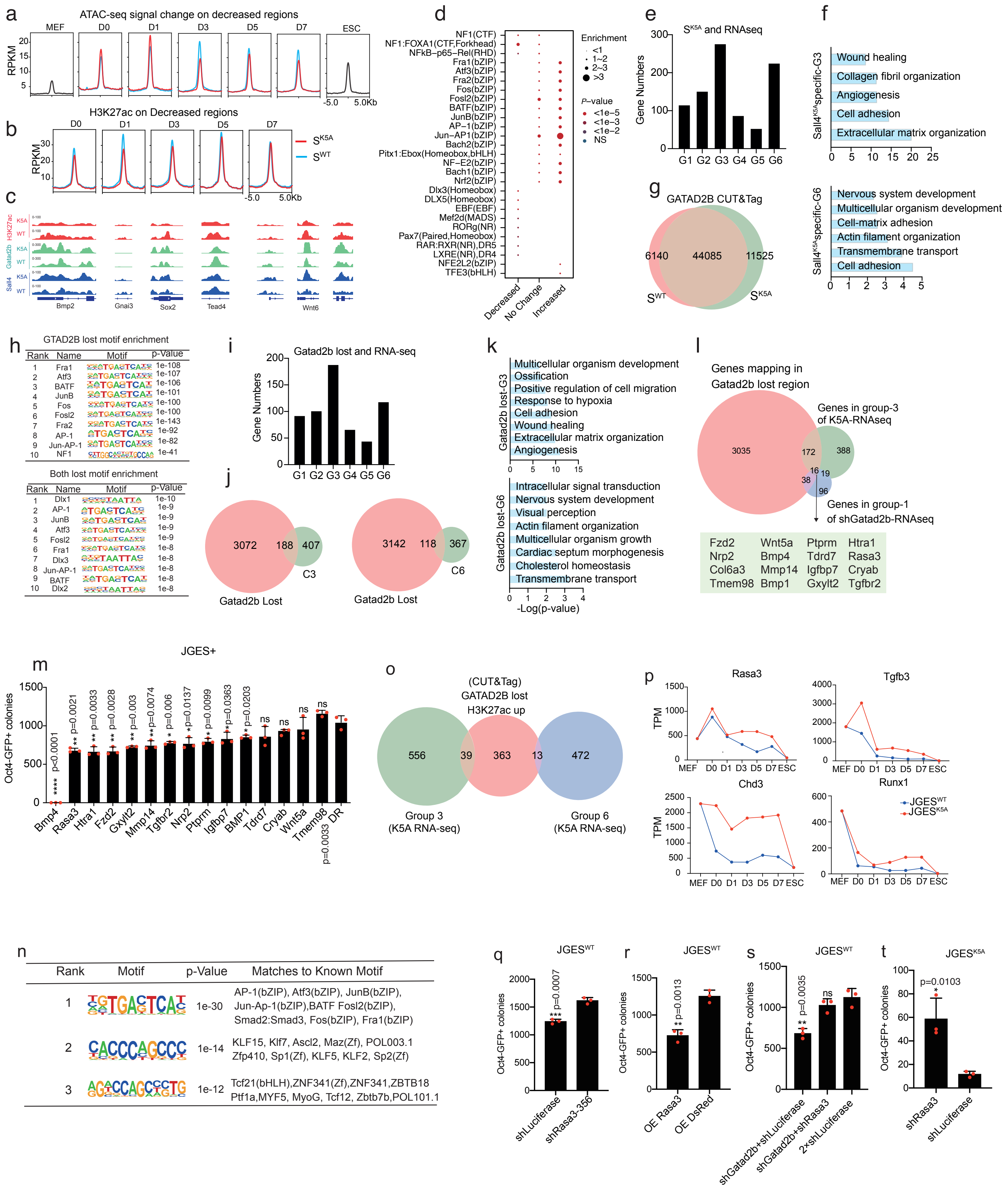

a
ATAC-seq signal change on decreased regions
MEF D0 D1 D3 D5 D7 ESC
RPKM
b
H3K27ac on Decreased regions
SK5A
SWT
c
Bmp2 Gnai3 Sox2 Tead4 Wnt6
d
Enrichment
P-value
Decreased
No Change
Increased
e
SK5A and RNAseq
Gene Numbers
G1 G2 G3 G4 G5 G6
f
Wound healing
Collagen fibril organization
Angiogenesis
Cell adhesion
Extracellular matrix organization
Nervous system development
Multicellular organism development
Cell-matrix adhesion
Actin filament organization
Transmembrane transport
Cell adhesion
g
GATAD2B CUT&Tag
6140 44085 11525
SWT SK5A
h
GTAD2B lost motif enrichment
Both lost motif enrichment
i
Gatad2b lost and RNA-seq
j
3072 188 407 C3
3142 118 367 C6
Gatad2b Lost
k
Multicellular organism development
Ossification
Positive regulation of cell migration
Response to hypoxia
Cell adhesion
Wound healing
Extracellular matrix organization
Angiogenesis
Intracellular signal transduction
Nervous system development
Visual perception
Actin filament organization
Multicellular organism growth
Cardiac septum morphogenesis
Cholesterol homeostasis
Transmembrane transport
-Log(p-value)
l
Genes mapping in Gatad2b lost region
Genes in group-3 of K5A-RNAseq
Genes in group-1 of shGatad2b-RNAseq
3035 172 388 16 19 38 96
Fzd2 Wnt5a Ptprm Htra1
Nrp2 Bmp4 Tdrd7 Rasa3
Col6a3 Mmp14 Igfbp7 Cryab
Tmem98 Bmp1 Gxylt2 Tgfbr2
m
JGES+
Oct4-GFP+ colonies
n
Rank Motif p-Value Matches to Known Motif
1 1e-30 AP-1(bZIP), Atf3(bZIP), JunB(bZIP), Jun-Ap-1(bZIP),BATF Fosl2(bZIP), Smad2:Smad3, Fos(bZIP), Fra1(bZIP)
2 1e-14 KLF15, Klf7, Ascl2, Maz(Zf), POL003.1 Zfp410, Sp1(Zf), KLF5, KLF2, Sp2(Zf)
3 1e-12 Tcf21(bHLH),ZNF341(Zf),ZNF341,ZBTB18 Ptf1a,MYF5, MyoG, Tcf12, Zbtb7b,POL101.1
o
(CUT&Tag) GATAD2B lost H3K27ac up
556 39 363 13 472
Group 3 (K5A RNA-seq)
Group 6 (K5A RNA-seq)
p
Rasa3 Tgfb3 Chd3 Runx1
TPM
JGESWT
JGESK5A
q JGESWT shLuciferase shRasa3-356
r JGESWT OE Rasa3 OE DsRed
s JGESWT shGatad2b+shLuciferase shGatad2b+shRasa3 2xshLuciferase
t JGESK5A shRasa3 shLuciferase p=0.0103

Supplementary Fig. 4 SALL4 K5A mutation fail to close the somatic chromatin

a, b,Pileup of ATAC-seq and H3K27ac signal of decreased regions in 3c for MEF, day0-7(JGE+Sall4^{WT}/Sall4^{K5A} reprogramming), and ESC. **c**, selected genomic views of H3K27ac (day1) for the indicated genes. **d**, Enriched motifs of regions in 3c. Hypergeometric test. **e**, Histogram showing the overlap of genes between Sall4^{K5A} specific annotated nearest genes (Fig.3a) and RNA-seq data (Fig.2i). **f**, Gene ontology (GO) analysis of the genes in cluster 3 and 6 (Fig S4e). The p-value in GO results were calculated by hypergeometric test. **g**, overlapping peaks on day 1 in JGES^{WT} or JGES^{K5A} reprogramming. **h**, Enriched motifs of both lost regions and Gatad2b lost regions in 3k. Hypergeometric test. **i**, **j**, overlap of genes between Gatad2b lost annotated nearest genes (Fig 3i) and RNA-seq data (Fig 2i). **k**, Gene ontology (GO) analysis of the genes (Fig S3j), **l**, overlapping genes between Gatad2b lost regions (Fig **3i)**, gene cluster 1 in Fig **1f** and gene cluster3 in Fig **2i**. The common genes in those three groups were listed in the box below venn map. **m**, Oct4-GFP positive colonies of overexpression factors in Fig S4l during JGES reprogramming at day7. Data are mean ± s.d., two-sided, unpaired t test; n = 3 independent experiments, ns > 0.05. *p< 0.05, **p < 0.01, ****p < 0.0001. **n**, Enriched motifs of the regions in Fig 3m. **o**, Venn diagram showing the overlapping genes between annotated nearest genes in Fig 3l and genes in cluster3/6 in Fig 2i. **p**, Line plot showing the expression changes of genes in Fig S4nl. **q**, **r**, Oct4-GFP positive colonies of knocking down and overexpressing of Rasa3 in JGES reprograming system at day7. Data are mean ± s.d., two-sided, unpaired t test; n = 3 independent experiments, ***p < 0.001. **s**, **t,** knocking down Rasa3 could rescue knocking down of Gatad2b in JGES reprograming. Data are mean ± s.d., two-sided, unpaired t test; n = 3 independent experiments, ***p < 0.001. Source data related to Fig s1m, q-t are provided as a Source Data file.

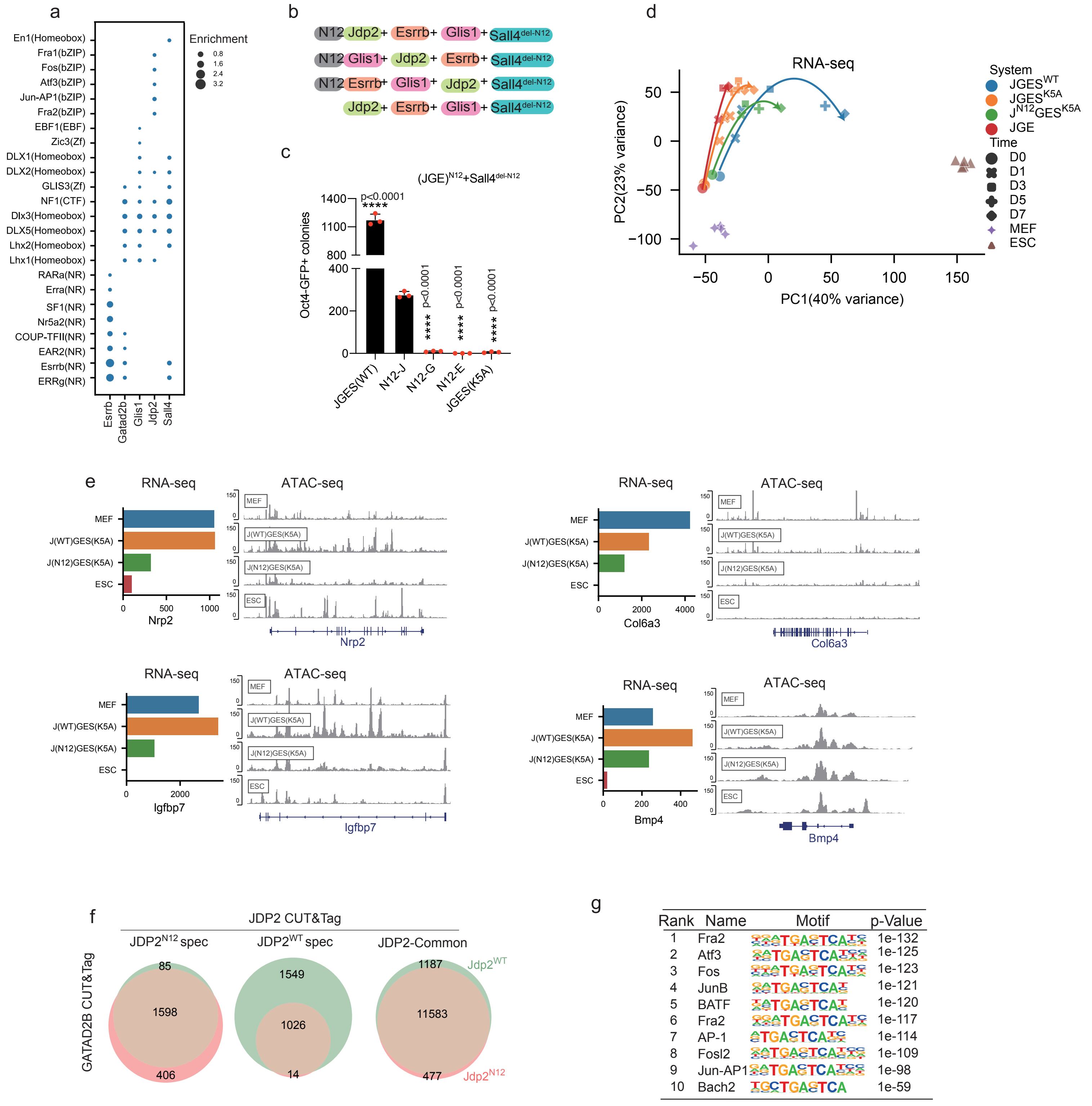
a
Enrichment
En1(Homeobox)
Fra1(bZIP)
Fos(bZIP)
Atf3(bZIP)
Jun-AP1(bZIP)
Fra2(bZIP)
EBF1(EBF)
Zic3(Zf)
DLX1(Homeobox)
DLX2(Homeobox)
GLIS3(Zf)
NF1(CTF)
Dlx3(Homeobox)
DLX5(Homeobox)
Lhx2(Homeobox)
Lhx1(Homeobox)
RARa(NR)
Erra(NR)
SF1(NR)
Nr5a2(NR)
COUP-TFII(NR)
EAR2(NR)
Esrrb(NR)
ERRg(NR)
Esrrb
Gatad2b
Glis1
Jdp2
Sall4
b
N12 Jdp2+ Esrrb+ Glis1+ Sall4del-N12
N12 Glis1+ Jdp2+ Esrrb+ Sall4del-N12
N12 Esrrb+ Glis1+ Jdp2+ Sall4del-N12
Jdp2+ Esrrb+ Glis1+ Sall4del-N12
c
(JGE)N12+Sall4del-N12
Oct4-GFP+ colonies
p<0.0001
JGES(WT)
N12-J
N12-G
N12-E
JGES(K5A)
d
RNA-seq
PC2(23% variance)
PC1(40% variance)
System
JGESWT
JGESK5A
JN12GESK5A
JGE
Time
D0
D1
D3
D5
D7
MEF
ESC
e
RNA-seq
ATAC-seq
MEF
J(WT)GES(K5A)
J(N12)GES(K5A)
ESC
Nrp2
Col6a3
Igfbp7
Bmp4
f
JDP2 CUT&Tag
JDP2N12 spec
JDP2WT spec
JDP2-Common
GATAD2B CUT&Tag
85
1598
406
1549
1026
14
1187
11583
477
Jdp2WT
Jdp2N12
g
Rank Name Motif p-Value
1 Fra2 1e-132
2 Atf3 1e-125
3 Fos 1e-123
4 JunB 1e-121
5 BATF 1e-120
6 Fra2 1e-117
7 AP-1 1e-114
8 Fosl2 1e-109
9 Jun-AP1 1e-98
10 Bach2 1e-59

Supplementary Fig. 5 Rescue of Sall4 N12 deletion by grafting N12 onto Jdp2

a, Dot plot showing the motifs enriched by Esrrb, Gatad2b, Glis1, Jdp2 and Sall4 on day 2 of reprogramming. **b**, Diagram showing grafting N12 individually to Jdp2, Esrrb, and Glis1 during reprogramming with Sall4^{delN12}. Jdp2^{N12}: (Jdp2^{N12}+Glis1+Esrrb+Sall4^{delN12}), Glis1^{N12}:(Jdp2+Glis1^{N12}+Esrrb+Sall4^{delN12}), Esrrb^{N12}:(Jdp2+Glis1+Esrrb^{N12}+Sall4^{delN12}). **c**, Numbers of Oct4 GFP positive iPS colonies induced by Jdp2^{N12}, Glis1^{N12}, and Esrrb^{N12}; $n = 3$ independent experiments, ****$p < 0.0001$. **d**, PCA of RNA-seq for MEF, JGES, JGE, JGE with K5A mut Sall4, GE with N12 delete Jdp2 and K5A mut Sall4 and ESC samples. Solid line with arrows represents one reprogramming sample at sequential time. **e**, selected genomic views of ATAC-seq data (right) are shown for the indicated genes in Jdp2$^{N12/WT}$ and Sall4^{K5A} reprogramming on day 1, MEF, and ESC with corresponding RNA expression (left). **f,** Venn diagram showing the overlap of GATAD2B binding sites on JDP2 binding sites. JDP2 CUT&Tag and GATAD2B CUT&Tag were performed in Jdp2^{WT} and Jdp2^{N12} condition. JDP2 binding sites were divided into three distinct categories: JDP2^{WT}-spec, JDP2-common, JDP2^{N12}-spec. **g**, Motif enrichment of overlap binding sites for GATAD2B in Jdp2^{N12} condition (406). Source data related to Fig s5c is provided as a Source Data file.
