# Supplementary material for: The NuRD complex cooperates with SALL4 to orchestrate reprogramming
Source: Nat Commun. 2023 May 18;14:2846. doi: 10.1038/s41467-023-38543-0 (PMC10199099; doi:10.1038/s41467-023-38543-0)
Supplement: Supplementary file 3 — Description of Additional Supplementary Files [file 41467_2023_38543_MOESM3_ESM.pdf]

Description of Additional Supplementary Files

File Name: Supplementary Data 1
Description: Raw data of chemical screening related to Figure s1c

File Name: Supplementary Data 2
Description: PCR primers for the detection of exogenous insertion of reprogramming factors related to Figure s1j

File Name: Supplementary Data 3
Description: Uncropped scans of gels related to Figure s1j

File Name: Supplementary Data 4
Description: Processed IP-MS data for Figure 1c, 2c, and 4c.

File Name: Supplementary Data 5
Description: shRNA oligos for the construction of pSuper-shRNA to knockdown the genes related to Figure s2a, s3e, s4q

File Name: Supplementary Data 6
Description: q-PCR primers for the detection knockdown efficiency of pSuper-shRNA related to Figure s2a, s3e, s4q
