# Supplementary material for: The NuRD complex cooperates with SALL4 to orchestrate reprogramming
Source: Nat Commun. 2023 May 18;14:2846. doi: 10.1038/s41467-023-38543-0 (PMC10199099; doi:10.1038/s41467-023-38543-0)
Supplement: Supplementary file 10 — Reporting Summary [file 41467_2023_38543_MOESM10_ESM.pdf]

nature portfolio

Corresponding author(s): Duanqing Pei

Last updated by author(s): Apr 18, 2023

Reporting Summary

Nature Portfolio wishes to improve the reproducibility of the work that we publish. This form provides structure for consistency and transparency in reporting. For further information on Nature Portfolio policies, see our Editorial Policies and the Editorial Policy Checklist.

Statistics

For all statistical analyses, confirm that the following items are present in the figure legend, table legend, main text, or Methods section.

n/a	Confirmed	
☐	☒	The exact sample size (*n*) for each experimental group/condition, given as a discrete number and unit of measurement
☐	☒	A statement on whether measurements were taken from distinct samples or whether the same sample was measured repeatedly
☐	☒	The statistical test(s) used AND whether they are one- or two-sided<br>*Only common tests should be described solely by name; describe more complex techniques in the Methods section.*
☒	☐	A description of all covariates tested
☒	☐	A description of any assumptions or corrections, such as tests of normality and adjustment for multiple comparisons
☐	☒	A full description of the statistical parameters including central tendency (e.g. means) or other basic estimates (e.g. regression coefficient) AND variation (e.g. standard deviation) or associated estimates of uncertainty (e.g. confidence intervals)
☒	☐	For null hypothesis testing, the test statistic (e.g. *F*, *t*, *r*) with confidence intervals, effect sizes, degrees of freedom and *P* value noted<br>*Give P values as exact values whenever suitable.*
☒	☐	For Bayesian analysis, information on the choice of priors and Markov chain Monte Carlo settings
☒	☐	For hierarchical and complex designs, identification of the appropriate level for tests and full reporting of outcomes
☒	☐	Estimates of effect sizes (e.g. Cohen's *d*, Pearson's *r*), indicating how they were calculated

Our web collection on statistics for biologists contains articles on many of the points above.

Software and code

Policy information about availability of computer code

Data collection	RNA-seq, ChIP-seq and ATAC-seq data are collected at home by using the specific commercial kit(Vazyme, NR611, TD901,TD501).
Data analysis	RNA-seq data were mapping to mm10 using RSEM(v. 1.2.28); ATAC-seq and ChIP-seq data data were mapped to the mm10 mouse genome assembly using bowtie2(v. 2.3.5.1). GO analysis for RNAseq: clusterProfiler(v. 3.14.3); Peaks calling: MACS2(v. 2.2.6) (for ChIPseq), Genrich(v. 0.6) (for ATACseq); Motif analysis, HOMER(v.4.11). Differential gene/peak analysis: DESeq2(v. 1.26.0), MAnorm(v. 1.3.0), diffbind(v. 2.14.0). sambamba(v. 0.6.6) was used to sort and remove duplicate reads with bam files. bigWig files were generated by deepTools(v. 3.5.1). Peak was annotated to gene loci by ChIPseeker(v. 3.20.1). Oct4-GFP positive clones number was counted by Image-J(v. 1.52).P-value of samples were calculated by Prism 6(v-6.01).

For manuscripts utilizing custom algorithms or software that are central to the research but not yet described in published literature, software must be made available to editors and reviewers. We strongly encourage code deposition in a community repository (e.g. GitHub). See the Nature Portfolio guidelines for submitting code & software for further information.

Data

Policy information about availability of data

All manuscripts must include a data availability statement. This statement should provide the following information, where applicable:

- Accession codes, unique identifiers, or web links for publicly available datasets
- A description of any restrictions on data availability
- For clinical datasets or third party data, please ensure that the statement adheres to our policy

The data supporting the conclusions of this Article, including ChIP–seq for H3K27ac, Sall4, Jdp2, Gatad2b, Esrrb and Glis1 are available at GEO under accession GSE199612. The ATAC-seq and RNA-seq data were from GSE199609 and GSE199613. The RNA-seq data of MEF and ES cells was obtained from GSE127927. Source Data for Figs 1, 2, 4 and Extended Data Figs 1-5 are provided with the manuscript. mouse genome assembly(mm10) data was downloaded from ensembl database.
https://www.ncbi.nlm.nih.gov/geo/query/acc.cgi?acc=GSE199614
https://www.ncbi.nlm.nih.gov/geo/query/acc.cgi

Research involving human participants, their data, or biological material

Policy information about studies with human participants or human data. See also policy information about sex, gender (identity/presentation), and sexual orientation and race, ethnicity and racism.

Reporting on sex and gender	*Use the terms sex (biological attribute) and gender (shaped by social and cultural circumstances) carefully in order to avoid confusing both terms. Indicate if findings apply to only one sex or gender; describe whether sex and gender were considered in study design; whether sex and/or gender was determined based on self-reporting or assigned and methods used. Provide in the source data disaggregated sex and gender data, where this information has been collected, and if consent has been obtained for sharing of individual-level data; provide overall numbers in this Reporting Summary. Please state if this information has not been collected. Report sex- and gender-based analyses where performed, justify reasons for lack of sex- and gender-based analysis.*
Reporting on race, ethnicity, or other socially relevant groupings	*Please specify the socially constructed or socially relevant categorization variable(s) used in your manuscript and explain why they were used. Please note that such variables should not be used as proxies for other socially constructed/relevant variables (for example, race/ethnicity should not be used as a proxy for socioeconomic status). Provide clear definitions of the relevant terms used, how they were provided (by the participants/respondents, the researchers, or third parties), and the method(s) used to classify people into the different categories (e.g. self-report, census or administrative data, social media data, etc.) Please provide details about how you controlled for confounding variables in your analyses.*
Population characteristics	*Describe the covariate-relevant population characteristics of the human research participants (e.g. age, genotypic information, past and current diagnosis and treatment categories). If you filled out the behavioural & social sciences study design questions and have nothing to add here, write "See above."*
Recruitment	*Describe how participants were recruited. Outline any potential self-selection bias or other biases that may be present and how these are likely to impact results.*
Ethics oversight	*Identify the organization(s) that approved the study protocol.*

Note that full information on the approval of the study protocol must also be provided in the manuscript.

Field-specific reporting

Please select the one below that is the best fit for your research. If you are not sure, read the appropriate sections before making your selection.

☒ Life sciences ☐ Behavioural & social sciences ☐ Ecological, evolutionary & environmental sciences

For a reference copy of the document with all sections, see nature.com/documents/nr-reporting-summary-flat.pdf

Life sciences study design

All studies must disclose on these points even when the disclosure is negative.

Sample size	we knockdown all the NuRD subunits and found Chd4, Gatad2b/2a are critical component for reprogramming. Then we detected how did they infect reprogramming. We made mutations for each amino acid resides N terminal of SALL4 and analysis their defection during reprogramming. The sample for each figure in this study has positive and negative control. No statistical method was used to predetermine sample size.
Data exclusions	No data were excluded from analysis.
Replication	All experiments were replicated at least three times, and data are shown as means with SEM.
Randomization	The authors thought there were no relevant for randomization to our study. No statistical method was used to predetermine sample size. No

Randomization	specific randomization or blinding protocols were used. Each experiment had positive and negative controls, and the sample size was carefully designed to support the conclusions
Blinding	*Describe whether the investigators were blinded to group allocation during data collection and/or analysis. If blinding was not possible, describe why OR explain why blinding was not relevant to your study.*

Behavioural & social sciences study design

All studies must disclose on these points even when the disclosure is negative.

Study description	*Briefly describe the study type including whether data are quantitative, qualitative, or mixed-methods (e.g. qualitative cross-sectional, quantitative experimental, mixed-methods case study).*
Research sample	*State the research sample (e.g. Harvard university undergraduates, villagers in rural India) and provide relevant demographic information (e.g. age, sex) and indicate whether the sample is representative. Provide a rationale for the study sample chosen. For studies involving existing datasets, please describe the dataset and source.*
Sampling strategy	*Describe the sampling procedure (e.g. random, snowball, stratified, convenience). Describe the statistical methods that were used to predetermine sample size OR if no sample-size calculation was performed, describe how sample sizes were chosen and provide a rationale for why these sample sizes are sufficient. For qualitative data, please indicate whether data saturation was considered, and what criteria were used to decide that no further sampling was needed.*
Data collection	*Provide details about the data collection procedure, including the instruments or devices used to record the data (e.g. pen and paper, computer, eye tracker, video or audio equipment) whether anyone was present besides the participant(s) and the researcher, and whether the researcher was blind to experimental condition and/or the study hypothesis during data collection.*
Timing	*Indicate the start and stop dates of data collection. If there is a gap between collection periods, state the dates for each sample cohort.*
Data exclusions	*If no data were excluded from the analyses, state so OR if data were excluded, provide the exact number of exclusions and the rationale behind them, indicating whether exclusion criteria were pre-established.*
Non-participation	*State how many participants dropped out/declined participation and the reason(s) given OR provide response rate OR state that no participants dropped out/declined participation.*
Randomization	*If participants were not allocated into experimental groups, state so OR describe how participants were allocated to groups, and if allocation was not random, describe how covariates were controlled.*

Ecological, evolutionary & environmental sciences study design

All studies must disclose on these points even when the disclosure is negative.

Study description	*Briefly describe the study. For quantitative data include treatment factors and interactions, design structure (e.g. factorial, nested, hierarchical), nature and number of experimental units and replicates.*
Research sample	*Describe the research sample (e.g. a group of tagged Passer domesticus, all Stenocereus thurberi within Organ Pipe Cactus National Monument), and provide a rationale for the sample choice. When relevant, describe the organism taxa, source, sex, age range and any manipulations. State what population the sample is meant to represent when applicable. For studies involving existing datasets, describe the data and its source.*
Sampling strategy	*Note the sampling procedure. Describe the statistical methods that were used to predetermine sample size OR if no sample-size calculation was performed, describe how sample sizes were chosen and provide a rationale for why these sample sizes are sufficient.*
Data collection	*Describe the data collection procedure, including who recorded the data and how.*
Timing and spatial scale	*Indicate the start and stop dates of data collection, noting the frequency and periodicity of sampling and providing a rationale for these choices. If there is a gap between collection periods, state the dates for each sample cohort. Specify the spatial scale from which the data are taken*
Data exclusions	*If no data were excluded from the analyses, state so OR if data were excluded, describe the exclusions and the rationale behind them, indicating whether exclusion criteria were pre-established.*
Reproducibility	*Describe the measures taken to verify the reproducibility of experimental findings. For each experiment, note whether any attempts to repeat the experiment failed OR state that all attempts to repeat the experiment were successful.*
Randomization	*Describe how samples/organisms/participants were allocated into groups. If allocation was not random, describe how covariates were controlled. If this is not relevant to your study, explain why.*
Blinding	*Describe the extent of blinding used during data acquisition and analysis. If blinding was not possible, describe why OR explain why blinding was not relevant to your study.*

Did the study involve field work? ☐ Yes ☐ No

Field work, collection and transport

Field conditions	*Describe the study conditions for field work, providing relevant parameters (e.g. temperature, rainfall).*
Location	*State the location of the sampling or experiment, providing relevant parameters (e.g. latitude and longitude, elevation, water depth).*
Access & import/export	*Describe the efforts you have made to access habitats and to collect and import/export your samples in a responsible manner and in compliance with local, national and international laws, noting any permits that were obtained (give the name of the issuing authority, the date of issue, and any identifying information).*
Disturbance	*Describe any disturbance caused by the study and how it was minimized.*

Reporting for specific materials, systems and methods

We require information from authors about some types of materials, experimental systems and methods used in many studies. Here, indicate whether each material, system or method listed is relevant to your study. If you are not sure if a list item applies to your research, read the appropriate section before selecting a response.

Materials & experimental systems

n/a	Involved in the study	
☐	☒	Antibodies
☐	☒	Eukaryotic cell lines
☒	☐	Palaeontology and archaeology
☐	☒	Animals and other organisms
☒	☐	Clinical data
☒	☐	Dual use research of concern
☒	☐	Plants

Methods

n/a	Involved in the study	
☐	☒	ChIP-seq
☒	☐	Flow cytometry
☒	☐	MRI-based neuroimaging

Antibodies

Antibodies used

SALL4 abcam ab29112, H3K27ac abcam ab4729, HDAC1 Cell Signaling Technology (10E2) Mouse mAb #5356, HDAC2 Cell Signaling Technology (3F3) Mouse mAb #5113, MTA1 Cell Signaling Technology (D40D1) XP® Rabbit mAb #5647, RBAP4 Cell Signaling Technology (V415) Antibody #6882, GATAD2B abcam Ab224391, FLAG-Tag MilliporeSigma F1804, HA-Tag Cell Signaling Technology (C29F4) 3724 , ESRRB R&D Systems PP-H6705-00. All the primary antibodies are diluted by 1:50 for cut-tag, and 1:1000 for Western Blot. Secondary antibodies were used as below, Goat anti-Rabbit IgG (H+L) Secondary Antibody, HRP (Invitrogen, 31460,1:5000 for Western Blot), Goat anti-Mouse IgG (H+L) Secondary Antibody, HRP(Invitrogen, 31430,1:10000 for Western Blot), Goat Anti-Rabbit IgG H&L(Abcam, Ab6702, 1:100 for cutandtag), Goat Anti-Mouse IgG H&L(Abcam, Ab6708, 1:100 for cutandtag).

Validation

SALL4 ab29112: Anti-Sall4 antibody - Abcam - CiteAb
H3K27ac ab4729: Anti-Histone H3 (acetyl K27) antibody - ChIP Grade - Abcam - CiteAb
HDAC1 (10E2) Mouse mAb #5356: (5356) HDAC1 (10E2) Mouse mAb - Cell Signaling Technology - CiteAb
HDAC2 (3F3) Mouse mAb #5113: (5113) HDAC2 (3F3) Mouse mAb - Cell Signaling Technology - CiteAb
MTA1 (D40D1) XP® Rabbit mAb #5647: (5647) MTA1 (D40D1) XP® Rabbit mAb - Cell Signaling Technology - CiteAb
RBAP4 (V415) Antibody #6882: (6882) RBAP46 (V415) Antibody - Cell Signaling Technology - CiteAb
GATAD2B Ab224391: (ab224391) Anti-GATAD2B antibody - Abcam - CiteAb
FLAG-Tag F1804: (F1804) Monoclonal ANTI-FLAG(R) M2 antibody produced in mouse - MilliporeSigma - CiteAb
HA-Tag (C29F4) Rabbit mAb: (3724) HA-Tag (C29F4) Rabbit mAb - Cell Signaling Technology - CiteAb
ESRRB PP-H6705-00: (PP-H6705-00) Human ERR beta/NR3B2 Antibody - R&D Systems - CiteAb
Goat Anti-Rabbit IgG H&L(Abcam, Ab6702):https://www.citeab.com/antibodies/4636511-ab6702-goat-anti-rabbit-igg-h-l?des=f9df39476fa23748
Goat Anti-Mouse IgG H&L(Abcam, Ab6708):https://www.citeab.com/antibodies/4636512-ab6708-goat-anti-mouse-igg-h-l?des=dda5ce303767c281
Goat anti-Mouse IgG (H+L)(31430) Secondary Antibody, HRP:https://www.citeab.com/antibodies/12179290-31430-goat-anti-mouse-igg-h-l-secondary-antibody?des=564f97edb78882c3
Goat anti-Rabbit IgG (H+L) (31460) Secondary Antibody, HRP:https://www.citeab.com/antibodies/12179302-31460-goat-anti-rabbit-igg-h-l-secondary-antibody?des=9be48fbf01a04749

Eukaryotic cell lines

Policy information about cell lines and Sex and Gender in Research

Cell line source(s)	MEF cells :MEFs were isolated from E13.5 mouse embryos regardless of sex from crossing male Oct4-GFP transgenic allele-carrying mice (CBA/CaJ 3 C57BL/6J) to 129S4/SvJaeJ female mice around 6-8 weeks old.<br>Platinum-E (Plat-E) is a potent retrovirus packaging cell line generated based on the 293T(Human Embryonic Kidney, HEK293) cell line. Conventional packaging constructs made use of the promoter of MuLV-LTR for expression of viral structural genes gag-pol and env, while our packaging constructs utilized the EF1α promoter, which is 100-fold more potent than the MuLV-LTR in 293T cells in combination with the Kozak's consensus sequence upstream of the initiation codon resulting in high expression of virus structural proteins in Plat-E cells.
Authentication	all the cell line uesed were authenticated
Mycoplasma contamination	all cell lines tested negative for mycoplasma
Commonly misidentified lines (See ICLAC register)	none

Palaeontology and Archaeology

Specimen provenance	*Provide provenance information for specimens and describe permits that were obtained for the work (including the name of the issuing authority, the date of issue, and any identifying information). Permits should encompass collection and, where applicable, export.*
Specimen deposition	*Indicate where the specimens have been deposited to permit free access by other researchers.*
Dating methods	*If new dates are provided, describe how they were obtained (e.g. collection, storage, sample pretreatment and measurement), where they were obtained (i.e. lab name), the calibration program and the protocol for quality assurance OR state that no new dates are provided.*

☐ Tick this box to confirm that the raw and calibrated dates are available in the paper or in Supplementary Information.

Ethics oversight	*Identify the organization(s) that approved or provided guidance on the study protocol, OR state that no ethical approval or guidance was required and explain why not.*

Note that full information on the approval of the study protocol must also be provided in the manuscript.

Animals and other research organisms

Policy information about studies involving animals; ARRIVE guidelines recommended for reporting animal research, and Sex and Gender in Research

Laboratory animals	OG2 transgenic mouse (CBA/CaJ x C57BL/6J) were purchased from the Jackson laboratories (Mouse strain datasheet: 004654). Animals were individually housed under a 12hr light/dark cycle and provided with food and water ad libitum.
Wild animals	none
Reporting on sex	none
Field-collected samples	none
Ethics oversight	Our studies followed the guidelines for the Care and Use of Laboratory Animals of the National Institutes of Health, and the protocols were approved by the Committee on the Ethics of Animal Experiments at the Guangzhou Institutes of Biomedicine and Health.

Note that full information on the approval of the study protocol must also be provided in the manuscript.

Clinical data

Policy information about clinical studies

All manuscripts should comply with the ICMJE guidelines for publication of clinical research and a completed CONSORT checklist must be included with all submissions.

Clinical trial registration	*Provide the trial registration number from ClinicalTrials.gov or an equivalent agency.*
Study protocol	*Note where the full trial protocol can be accessed OR if not available, explain why.*
Data collection	*Describe the settings and locales of data collection, noting the time periods of recruitment and data collection.*

Outcomes	*Describe how you pre-defined primary and secondary outcome measures and how you assessed these measures.*

Dual use research of concern

Policy information about dual use research of concern

Hazards

Could the accidental, deliberate or reckless misuse of agents or technologies generated in the work, or the application of information presented in the manuscript, pose a threat to:

No	Yes	
☐	☐	Public health
☐	☐	National security
☐	☐	Crops and/or livestock
☐	☐	Ecosystems
☐	☐	Any other significant area

Experiments of concern

Does the work involve any of these experiments of concern:

No	Yes	
☒	☐	Demonstrate how to render a vaccine ineffective
☐	☐	Confer resistance to therapeutically useful antibiotics or antiviral agents
☐	☐	Enhance the virulence of a pathogen or render a nonpathogen virulent
☐	☐	Increase transmissibility of a pathogen
☐	☐	Alter the host range of a pathogen
☐	☐	Enable evasion of diagnostic/detection modalities
☐	☐	Enable the weaponization of a biological agent or toxin
☐	☐	Any other potentially harmful combination of experiments and agents

Plants

Seed stocks	*Report on the source of all seed stocks or other plant material used. If applicable, state the seed stock centre and catalogue number. If plant specimens were collected from the field, describe the collection location, date and sampling procedures.*
Novel plant genotypes	*Describe the methods by which all novel plant genotypes were produced. This includes those generated by transgenic approaches, gene editing, chemical/radiation-based mutagenesis and hybridization. For transgenic lines, describe the transformation method, the number of independent lines analyzed and the generation upon which experiments were performed. For gene-edited lines, describe the editor used, the endogenous sequence targeted for editing, the targeting guide RNA sequence (if applicable) and how the editor was applied.*
Authentication	*Describe any authentication procedures for each seed stock used or novel genotype generated. Describe any experiments used to assess the effect of a mutation and, where applicable, how potential secondary effects (e.g. second site T-DNA insertions, mosiacism, off-target gene editing) were examined.*

ChIP-seq

Data deposition

☒ Confirm that both raw and final processed data have been deposited in a public database such as GEO.

☒ Confirm that you have deposited or provided access to graph files (e.g. BED files) for the called peaks.

Data access links<br>*May remain private before publication.*	https://www.ncbi.nlm.nih.gov/geo/query/acc.cgi?acc=GSE199614
Files in database submission	1)Raw Data:<br>J(N12)GES(K5A)-D1-JDP2-3F_1.fastq.gz<br>J(N12)GES(K5A)-D1-JDP2-3F_2.fastq.gz<br>JGES(K5A)-D1-Gatad2b_1.fastq.gz<br>JGES(K5A)-D1-Gatad2b_2.fastq.gz<br>JGES(K5A)-D1-H3K27ac-2_1.fastq.gz

JGES(K5A)-D1-H3K27ac-2_2.fastq.gz
JGES(K5A)-D1-H3K27ac_1.fastq.gz
JGES(K5A)-D1-H3K27ac_2.fastq.gz
JGES(K5A)-D1-JDP2-3F_1.fastq.gz
JGES(K5A)-D1-JDP2-3F_2.fastq.gz
JGES(K5A)-D1-Sall4-3Flag_1.fastq.gz
JGES(K5A)-D1-Sall4-3Flag_2.fastq.gz
JGES-D1-Gatad2b_1.fastq.gz
JGES-D1-Gatad2b_2.fastq.gz
JGES-D1-H3K27ac_1.fastq.gz
JGES-D1-H3K27ac_2.fastq.gz
JGES-D1-Sall4-3Flag_1.fastq.gz
JGES-D1-Sall4-3Flag_2.fastq.gz
JGES-D2-Esrrb_1.fastq.gz
JGES-D2-Esrrb_2.fastq.gz
JGES-D2-Gatad2b_1.fastq.gz
JGES-D2-Gatad2b_2.fastq.gz
JGES-D2-Glis1-HA_1.fastq.gz
JGES-D2-Glis1-HA_2.fastq.gz
JGES-D2-Jdp2-3Flag_1.fastq.gz
JGES-D2-Jdp2-3Flag_2.fastq.gz
JGES-D2-Sall4_1.fastq.gz
JGES-D2-Sall4_2.fastq.gz
J(N12)GES(K5A)-D1-Gatad2b_1.fastq.gz
J(N12)GES(K5A)-D1-Gatad2b_2.fastq.gz
JGES(K5A)-D0-H3K27ac_1.fastq.gz
JGES(K5A)-D0-H3K27ac_2.fastq.gz
JGES(K5A)-D1-Gatad2b_1.fastq.gz
JGES(K5A)-D1-Gatad2b_2.fastq.gz
JGES(K5A)-D1-H3K27ac_1.fastq.gz
JGES(K5A)-D1-H3K27ac_2.fastq.gz
JGES(K5A)-D3-H3K27ac_1.fastq.gz
JGES(K5A)-D3-H3K27ac_2.fastq.gz
JGES(K5A)-D5-H3K27ac_1.fastq.gz
JGES(K5A)-D5-H3K27ac_2.fastq.gz
JGES(K5A)-D7-H3K27ac_1.fastq.gz
JGES(K5A)-D7-H3K27ac_2.fastq.gz
JGES-D0-H3K27ac_1.fastq.gz
JGES-D0-H3K27ac_2.fastq.gz
JGES-D1-H3K27ac_1.fastq.gz
JGES-D1-H3K27ac_2.fastq.gz
JGES-D3-H3K27ac_1.fastq.gz
JGES-D3-H3K27ac_2.fastq.gz
JGES-D5-H3K27ac_1.fastq.gz
JGES-D5-H3K27ac_2.fastq.gz
JGES-D7-H3K27ac_1.fastq.gz
JGES-D7-H3K27ac_2.fastq.gz

2)Processed Data:
J(N12)GES(K5A)-D1-JDP2-3F.bw
JGES(K5A)-D1-Gatad2b.bw
JGES(K5A)-D1-H3K27ac-2.bw
JGES(K5A)-D1-H3K27ac.bw
JGES(K5A)-D1-JDP2-3F.bw
JGES(K5A)-D1-Sall4-3f.bw
JGES-D1-Gatad2b.bw
JGES-D1-H3K27ac.bw
JGES-D1-Sall4-3f.bw
JGES-D2-Esrrb.bw
JGES-D2-Gatad2b.bw
JGES-D2-HA-Glis1.bw
JGES-D2-Jdp2-3Flag.bw
JGES-D2-Sall4.bw
J(N12)GES(K5A)-D1-Gatad2b.bw
JGES(K5A)-D0-H3K27ac.bw
JGES(K5A)-D1-Gatad2b.bw
JGES(K5A)-D1-H3K27ac.bw
JGES(K5A)-D3-H3K27ac.bw
JGES(K5A)-D5-H3K27ac.bw
JGES(K5A)-D7-H3K27ac.bw
JGES-D0-H3K27ac.bw
JGES-D1-H3K27ac.bw
JGES-D3-H3K27ac.bw
JGES-D5-H3K27ac.bw
JGES-D7-H3K27ac.bw

Genome browser session (e.g. UCSC)	no longer applicable

Methodology

Replicates	All data have only one repeat, except for H3K27AC on day1. JGES-D1-H3k27ac has two repeats and JGES (K5A)-D1-H3K27ac has three repeats
Sequencing depth	J(N12)GES(K5A)-D1-JDP2-3F_1.fastq.gz 60958521 32308394 150bp paired-end<br>J(N12)GES(K5A)-D1-JDP2-3F_2.fastq.gz 60958521 32520641 150bp paired-end<br>JGES(K5A)-D1-Gatad2b_1.fastq.gz 41992742 8860935 150bp paired-end<br>JGES(K5A)-D1-Gatad2b_2.fastq.gz 41992742 12245604 150bp paired-end<br>JGES(K5A)-D1-H3K27ac-2_1.fastq.gz 50277205 37764330 150bp paired-end<br>JGES(K5A)-D1-H3K27ac-2_2.fastq.gz 50277205 39061082 150bp paired-end<br>JGES(K5A)-D1-H3K27ac_1.fastq.gz 40513410 29004592 150bp paired-end<br>JGES(K5A)-D1-H3K27ac_2.fastq.gz 40513410 29528897 150bp paired-end<br>JGES(K5A)-D1-JDP2-3F_1.fastq.gz 51919211 14922755 150bp paired-end<br>JGES(K5A)-D1-JDP2-3F_2.fastq.gz 51919211 14677417 150bp paired-end<br>JGES(K5A)-D1-Sall4-3Flag_1.fastq.gz 47692277 28876269 150bp paired-end<br>JGES(K5A)-D1-Sall4-3Flag_2.fastq.gz 47692277 31535695 150bp paired-end<br>JGES-D1-Gatad2b_1.fastq.gz 34829331 11463246 150bp paired-end<br>JGES-D1-Gatad2b_2.fastq.gz 34829331 15903571 150bp paired-end<br>JGES-D1-H3K27ac_1.fastq.gz 27644065 21333734 150bp paired-end<br>JGES-D1-H3K27ac_2.fastq.gz 27644065 21621061 150bp paired-end<br>JGES-D1-Sall4-3Flag_1.fastq.gz 50722397 22446497 150bp paired-end<br>JGES-D1-Sall4-3Flag_2.fastq.gz 50722397 26535968 150bp paired-end<br>JGES-D2-Esrrb_1.fastq.gz 31506325 19638516 150bp paired-end<br>JGES-D2-Esrrb_2.fastq.gz 31506325 20211937 150bp paired-end<br>JGES-D2-Gatad2b_1.fastq.gz 33022152 22755667 150bp paired-end<br>JGES-D2-Gatad2b_2.fastq.gz 33022152 23459743 150bp paired-end<br>JGES-D2-Glis1-HA_1.fastq.gz 35413918 7753934 150bp paired-end<br>JGES-D2-Glis1-HA_2.fastq.gz 35413918 10739030 150bp paired-end<br>JGES-D2-Jdp2-3Flag_1.fastq.gz 29178082 10896282 150bp paired-end<br>JGES-D2-Jdp2-3Flag_2.fastq.gz 29178082 12128866 150bp paired-end<br>JGES-D2-Sall4_1.fastq.gz 36676627 26667158 150bp paired-end<br>JGES-D2-Sall4_2.fastq.gz 36676627 27129561 150bp paired-end<br>J(N12)GES(K5A)-D1-Gatad2b_1 18310843 4973054 150bp paired-end<br>J(N12)GES(K5A)-D1-Gatad2b_2 18310843 6084666 150bp paired-end<br>JGES(K5A)-D0-H3K27ac_1 17535013 10717831 150bp paired-end<br>JGES(K5A)-D0-H3K27ac_2 17535013 11146549 150bp paired-end<br>JGES(K5A)-D1-Gatad2b_1 12520491 3933263 150bp paired-end<br>JGES(K5A)-D1-Gatad2b_2 12520491 4413789 150bp paired-end<br>JGES(K5A)-D1-H3K27ac_1 16002374 9666304 150bp paired-end<br>JGES(K5A)-D1-H3K27ac_2 16002374 10237828 150bp paired-end<br>JGES(K5A)-D3-H3K27ac_1 16557578 10868268 150bp paired-end<br>JGES(K5A)-D3-H3K27ac_2 16557578 11369884 150bp paired-end<br>JGES(K5A)-D5-H3K27ac_1 17748444 9094126 150bp paired-end<br>JGES(K5A)-D5-H3K27ac_2 17748444 9870438 150bp paired-end<br>JGES(K5A)-D7-H3K27ac_1 19321170 11455275 150bp paired-end<br>JGES(K5A)-D7-H3K27ac_2 19321170 12354684 150bp paired-end<br>JGES-D0-H3K27ac_1 14830496 9816014 150bp paired-end<br>JGES-D0-H3K27ac_2 14830496 9944216 150bp paired-end<br>JGES-D1-H3K27ac_1 13516396 8601152 150bp paired-end<br>JGES-D1-H3K27ac_2 13516396 8816024 150bp paired-end<br>JGES-D3-H3K27ac_1 15214328 10641085 150bp paired-end<br>JGES-D3-H3K27ac_2 15214328 11204185 150bp paired-end<br>JGES-D5-H3K27ac_1 20608867 9588095 150bp paired-end<br>JGES-D5-H3K27ac_2 20608867 10507272 150bp paired-end<br>JGES-D7-H3K27ac_1 17253890 11482813 150bp paired-end<br>JGES-D7-H3K27ac_2 17253890 11975833 150bp paired-end
Antibodies	Anti GATAD2B (Abcam, ab224391,1:50), anti-FLAG (Sigma Aldrich, F1804,1:50), anti SALL4 (Abcam, ab29112,1:50), anti-H3K27ac (Abcam ab4729,1:50), anti FLAG-Tag (for JDP2) (MilliporeSigma F1804, 1:50), anti HA-Tag (for Glis1) (Cell Signaling Technology C29F4,1:50) were used.Secondary antibodies Goat Anti-Rabbit IgG H&L(Abcam, Ab6702, 1:100), Goat Anti-Mouse IgG H&L (Abcam, Ab6708, 1:100) were used.
Peak calling parameters	Peaks were identified using MACS2 with default paired-end parameter setting
Data quality	We evaluated the data quality by track view
Software	Bowtie2 (v. 2.3.5.1), MACS2 (v. 2.2.6), Deeptools (v. 3.5.1), DiffBind (v. 2.1.4), MAnorm (v. 1.3.0), HOMER (v. 4.1.1), sambamba(v. 0.6.6)

Flow Cytometry

Plots

Confirm that:

- [ ] The axis labels state the marker and fluorochrome used (e.g. CD4-FITC).
- [ ] The axis scales are clearly visible. Include numbers along axes only for bottom left plot of group (a 'group' is an analysis of identical markers).
- [ ] All plots are contour plots with outliers or pseudocolor plots.
- [ ] A numerical value for number of cells or percentage (with statistics) is provided.

Methodology

Sample preparation	℃
Instrument	*Identify the instrument used for data collection, specifying make and model number.*
Software	*Describe the software used to collect and analyze the flow cytometry data. For custom code that has been deposited into a community repository, provide accession details.*
Cell population abundance	*Describe the abundance of the relevant cell populations within post-sort fractions, providing details on the purity of the samples and how it was determined.*
Gating strategy	*Describe the gating strategy used for all relevant experiments, specifying the preliminary FSC/SSC gates of the starting cell population, indicating where boundaries between "positive" and "negative" staining cell populations are defined.*

- [ ] Tick this box to confirm that a figure exemplifying the gating strategy is provided in the Supplementary Information.

Magnetic resonance imaging

Experimental design

Design type	*Indicate task or resting state; event-related or block design.*
Design specifications	*Specify the number of blocks, trials or experimental units per session and/or subject, and specify the length of each trial or block (if trials are blocked) and interval between trials.*
Behavioral performance measures	*State number and/or type of variables recorded (e.g. correct button press, response time) and what statistics were used to establish that the subjects were performing the task as expected (e.g. mean, range, and/or standard deviation across subjects).*

Acquisition

Imaging type(s)	*Specify: functional, structural, diffusion, perfusion.*
Field strength	*Specify in Tesla*
Sequence & imaging parameters	*Specify the pulse sequence type (gradient echo, spin echo, etc.), imaging type (EPI, spiral, etc.), field of view, matrix size, slice thickness, orientation and TE/TR/flip angle.*
Area of acquisition	*State whether a whole brain scan was used OR define the area of acquisition, describing how the region was determined.*

Diffusion MRI ☐ Used ☐ Not used

Preprocessing

Preprocessing software	*Provide detail on software version and revision number and on specific parameters (model/functions, brain extraction, segmentation, smoothing kernel size, etc.).*
Normalization	*If data were normalized/standardized, describe the approach(es): specify linear or non-linear and define image types used for transformation OR indicate that data were not normalized and explain rationale for lack of normalization.*
Normalization template	*Describe the template used for normalization/transformation, specifying subject space or group standardized space (e.g. original Talairach, MNI305, ICBM152) OR indicate that the data were not normalized.*
Noise and artifact removal	*Describe your procedure(s) for artifact and structured noise removal, specifying motion parameters, tissue signals and physiological signals (heart rate, respiration).*

Volume censoring | *Define your software and/or method and criteria for volume censoring, and state the extent of such censoring.*

Statistical modeling & inference

Model type and settings | *Specify type (mass univariate, multivariate, RSA, predictive, etc.) and describe essential details of the model at the first and second levels (e.g. fixed, random or mixed effects; drift or auto-correlation).*

Effect(s) tested | *Define precise effect in terms of the task or stimulus conditions instead of psychological concepts and indicate whether ANOVA or factorial designs were used.*

Specify type of analysis: ☐ Whole brain ☐ ROI-based ☐ Both

Statistic type for inference | *Specify voxel-wise or cluster-wise and report all relevant parameters for cluster-wise methods.*

(See Eklund et al. 2016)

Correction | *Describe the type of correction and how it is obtained for multiple comparisons (e.g. FWE, FDR, permutation or Monte Carlo).*

Models & analysis

n/a	Involved in the study
☐	☐ Functional and/or effective connectivity
☐	☐ Graph analysis
☐	☐ Multivariate modeling or predictive analysis

Functional and/or effective connectivity | *Report the measures of dependence used and the model details (e.g. Pearson correlation, partial correlation, mutual information).*

Graph analysis | *Report the dependent variable and connectivity measure, specifying weighted graph or binarized graph, subject- or group-level, and the global and/or node summaries used (e.g. clustering coefficient, efficiency, etc.).*

Multivariate modeling and predictive analysis | *Specify independent variables, features extraction and dimension reduction, model, training and evaluation metrics.*
